# Comparison of microbial communities and the profile of sulfate-reducing bacteria in patients with ulcerative colitis and their association with bowel diseases: a pilot study

**DOI:** 10.15698/mic2024.03.817

**Published:** 2024-03-14

**Authors:** Ivan Kushkevych, Kristýna Martínková, Lenka Mráková, Francesco Giudici, Simone Baldi, David Novak, Márió Gajdács, Monika Vítězová, Dani Dordevic, Amedeo Amedei, Simon K.-M. R. Rittmann

**Affiliations:** 1 Department of Experimental Biology, Faculty of Science, Masaryk University, 62500 Brno, Czech Republic.; 2 Department of Experimental and Clinical Medicine, University of Florence, 50134 Florence, Italy.; 3 Department of Biochemistry, Faculty of Science, Masaryk University, 62500 Brno, Czech Republic.; 4 Department of Oral Biology and Experimental Dental Research, Faculty of Dentistry, University of Szeged, 6720 Szeged, Hungary.; 5 Department of Plant Origin Food Sciences, Faculty of Veterinary Hygiene and Ecology, University of Veterinary Sciences Brno, Palackého tř. 1946/1, 612 42 Brno, Czech Republic.; 6 Department of Functional and Evolutionary Ecology, Archaea Physiology & Biotechnology Group, Universität Wien, 1030 Wien, Austria.

**Keywords:** gut microbiota, ulcerative colitis, gut dysbiosis, sulfate-reducing bacteria, inflammatory bowel disease, 16S rRNA gene sequencing

## Abstract

Considerable evidence has accumulated regarding the molecular relationship between gut microbiota (GM) composition and the onset (clinical presentation and prognosis of ulcerative colitis (UC)). In addition, it is well documented that short-chain fatty acid (SCFA)-producing bacteria may play a fundamental role in maintaining an anti-inflammatory intestinal homeostasis, but sulfate- and sulfite reducing bacteria may be responsible for the production of toxic metabolites, such as hydrogen sulfide and acetate. Hence, the present study aimed to assess the GM composition – focusing on sulfate-reducing bacteria (SRB) – in patients with severe, severe-active and moderate UC. Each one of the six enrolled patients provided two stool samples in the following way: one sample was cultivated in a modified SRB-medium before 16S rRNA sequencing and the other was not cultivated. Comparative phylogenetic analysis was conducted on each sample. Percentage of detected gut microbial genera showed considerable variation based on the patients' disease severity and cultivation in the SRB medium. In detail, samples without cultivation from patients with moderate UC showed a high abundance of the genera *Bacteroides*, *Bifidobacterium* and *Ruminococcus,* but after SRB cultivation, the dominant genera were *Bacteroides*, *Klebsiella* and *Bilophila*. On the other hand, before SRB cultivation, the main represented genera in patients with severe UC were *Escherichia-Shigella*, *Proteus*, *Methanothermobacter* and *Methanobacterium*. However, after incubation in the SRB medium *Bacteroides, Proteus, Alistipes* and *Lachnoclostridium* were predominant. Information regarding GM compositional changes in UC patients may aid the development of novel therapeutic strategies (e.g., probiotic preparations containing specific bacterial strains) to counteract the mechanisms of virulence of harmful bacteria and the subsequent inflammatory response that is closely related to the pathogenesis of inflammatory bowel diseases.

## INTRODUCTION

Inflammatory bowel diseases (IBD), mainly represented as Crohn's disease (CD) and ulcerative colitis (UC), are the most common inflammatory intestinal diseases worldwide, characterized by intermittent chronic inflammation of the gastrointestinal tract [1–3]. In contrast to CD – which may affect any part of the intestinal tract – UC causes only localized damage in the colon and the rectum [4]. This chronic disease is manifested by a dysregulated inflammatory process and an altered immune response to yet undefined environmental factors (in genetically predisposed individuals) [5]. As a result, UC patients present inflammatory reactions, erosions of the colonic wall and associated bleeding, thus, the most common UC clinical symptoms include diarrhea, abdominal pain, rectal bleeding and weight loss [6, 7]. In addition, the accumulation of intestinal contents may lead to the thickening of the intestinal wall; this condition is called a toxic megacolon, and may be responsible for severe, life-threatening complications [8].

With the emergence of next-generation sequencing (NGS) technologies, considerable evidence has accumulated regarding the relationship between gut microbiota (GM) composition and the onset, clinical presentation and prognosis of numerous chronic illnesses, including UC [9, 10]. While the GM, primarily composed of billions of bacterial species, subspecies, and biotypes, plays a crucial role in maintaining host homeostasis [11–16], disease occurrence can also be significantly influenced by environmental factors and genetic predisposition [17]. Significant differences in the representation of individual strains were found in UC patients, with a notable increase in the number of *Pseudomonatoda* (previously: *Proteobacteria*), a phylum including SRB [18–20].

Furthermore, modifications of the GM structure in UC patients have been often associated with alterations in microbial-derived metabolite production [21–26]. For instance, the levels of beneficial short-chain fatty acid (SCFA)-producing bacteria are typically reduced in both mucosal and fecal samples of IBD patients, compared to healthy individuals [27–30].

Moreover, increased concentrations of some bacteria are associated with a higher production of toxic metabolites that favor the onset and progression of intestinal diseases. For example, sulfate-reducing bacteria (SRB) are anaerobic microorganisms able to produce, by dissimilatory sulfate reduction, hydrogen sulfide (H_2_S), a highly toxic compound to all living organisms [23, 24, 31–35]. Recently, it has also been hypothesized that H_2_S is involved in the process of bowel inflammation and UC development, due to its ability to increase mucosal permeability and to block butyrate metabolism [22, 33, 36, 37]. Additionally, it has been recently documented that SRB can also produce biofilm in the gut, along with other pathogenic species (e.g., *Bacteroides* spp., *Pseudomonas* spp., *Clostridium* spp. and *Escherichia* spp.) and penetrate the blood vessels after damaging the intestinal epithelium [34]. In this context, the objective of this study was to evaluate the composition of the GM in patients with varying degrees of UC severity and activity: i) severe, ii) severe in an active state, and iii) moderate UC. Specifically, our focus was on cultivating SRB in a modified Postgate medium to assess their influence on altering intestinal communities.

## RESULTS

### Enrolled patients

Six patients with UC, comprising five males and one female, were enrolled in the study. The mean age of the participants was 52 years, ranging from 26 to 80 years. Clinical characteristics corresponding to these UC patients are reported in **Table 1**. Four patients were affected by severe UC and two (samples 5 and 6) were suffering from a moderate form of UC and five out of six patients showed a Mayo score of 12. In addition, only one patient (sample 5) reported both a relapsing active disease refractory to steroids and a family history of IBD.

**Table 1.** Clinical features of the enrolled patients.

**ID**	UC1	UC2	UC3	UC4	UC5	UC6
**Age**	63	54	34	F	80	57
**Gender**	M	M	M	26	M	M
**Smoking**	No	No	Yes	No	Ex-smoker	No
**Familiar history for IBD**	No	No	No	No	Yes	No
**Intestinal inflammation**	Acute severe disease	Acute severe disease	Acute severe disease	Acute severe disease	Relapsing active disease refractory to steroids	Acute severe disease
**Disease Stage**	Severe	Severe	Severe	Severe	Moderate	Moderate
**Mayo Score**	12	12	12	12	10	12
**SCCAI Score**	10	14	13	13	9	13
**Baron score**	3	3	3	3	3	3
**Medical history**	Nonalcoholic steatohepatitis	.	Allergic asthma	-	Chronic Obstructive Pulmonary Disease	Type 2 diabetes, hypothyroidism, post-traumatic seizures
**UC drugs**	Corticosteroids 40mgs during the previous week	Corticosteroids 40mgs during the previous week	Corticosteroids 40mgs during the previous week	Corticosteroids 40mgs during the previous week	Topical and oral 5-aminosalicylic acid	Corticosteroids 40mgs during the previous week

UC: ulcerative colitis, M: male, F: female, SCCAI: Simple Clinical Colitis Activity Index

### Bacterial genera identified in the GM of severe UC patients

**Figure 1:**
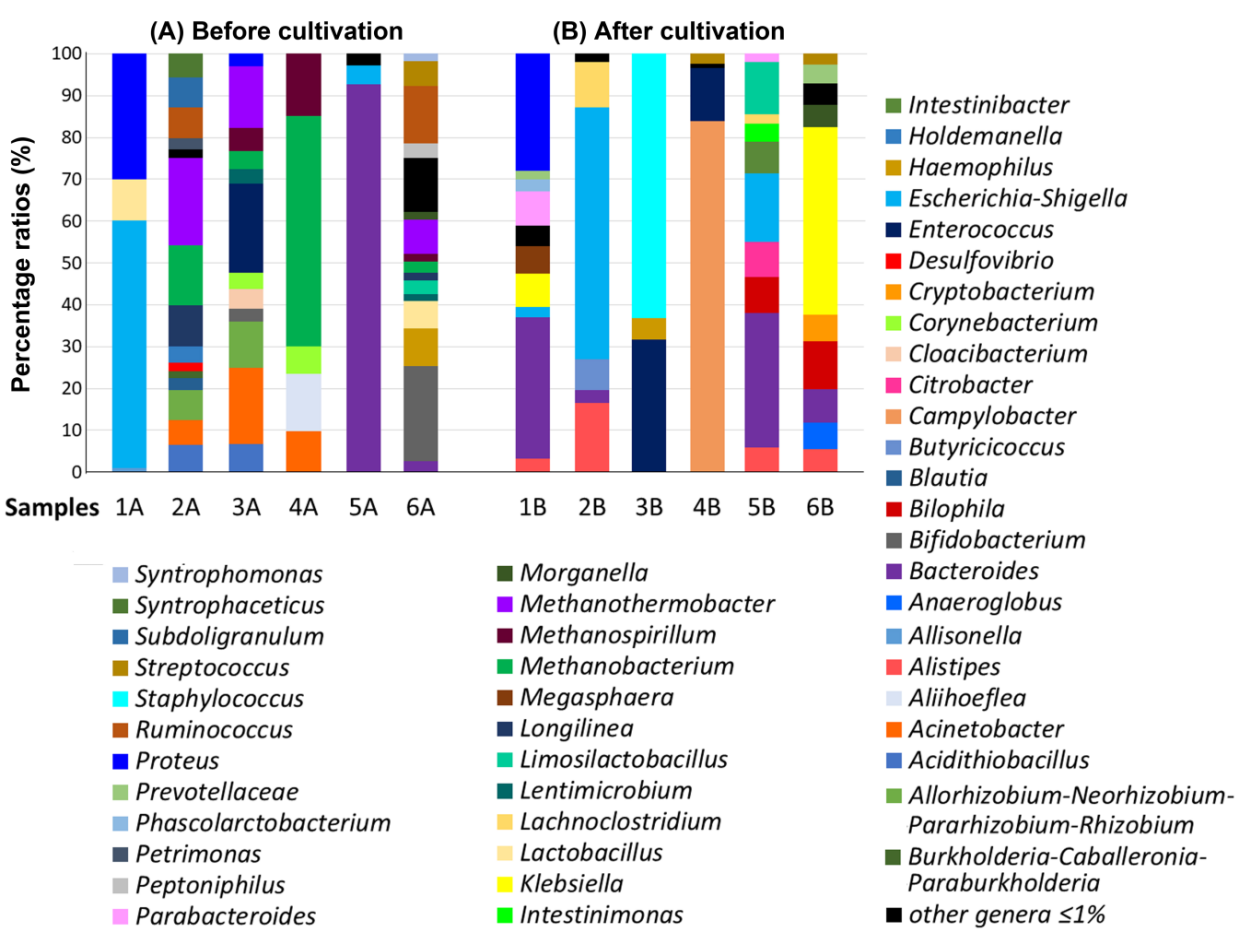
FIGURE 1: Stacked bar plots reporting the percentage of the microbial genera identified in the GM of severe UC patients. **(A)** Sequencing results of uncultivated stool samples, **(B)** sequencing results of stool samples after cultivation in SRB medium.

The percentage of individual genera in the GM of severe UC patients (samples 1, 2) was different both before cultivation as well as after cultivation in the SRB medium (**Figure 1**). The intestinal genera diversity in patient 1 (sample 1A) included bacterial species of only three main genera: *Escherichia-Shigella* (59%), *Proteus* (30%), *Lactobacillus* (10%). All other genera were detected at less than 1% (**Figure 1**). Inoculation of the sample into SRB medium and five days of cultivation led to bacterial compositional changes. We observed a dominance of *Bacteroides* species (34%) followed by the *Proteus* genus (28%). The percentage of *Proteus* spp. did not change significantly compared to the sample without cultivation (**Figure 1**). Percentage of other genera (*Klebsiella, Megasphaera, Escherichia-Shigella, Parabacteroides, Phascolarctobacterium, Prevotellaceae* NK3B31 group, *Alistipes*, *Pseudoramibacter, Paraprevotella, Solobacterium, Bilophila* and *Barnesiella*) in sample 1B after cultivation were less than or equal to 8%.

The representation of genera in the sample without cultivation taken from patient 2 (sample 2A) was more differentiated than in the sample after culture (sample 2B). The dominant genera in sample 2A were: *Methanothermobacter* (21%), *Methanobacterium* (14%) and *Longilinea* (10%). Other genera, including *Allorhizobium-Neorhizobium-Pararhizobium-Rhizobium, Ruminococcus, Subdoligranulum, Holdemanella, Acidithiobacillus, Acinetobacter, Syntrophaceticus, Blautia, Petrimonas, Desulfovibrio, Burkholderia-Caballeronia-Paraburkholderia, Porphyromonas* and *Actinomyces* were represented in percentage by less than 8%. The representation of genera in sample 2B exhibited less diversity compared to sample 2A, with *Escherichia-Shigella* (61%) identified as the predominant genus. Other relatively dominant genera were *Alistipes* (17%) and *Lachnoclostridium* (11%). *Butyricicoccus* (7%) and *Bacteroides* (3%) were also detected, while under-represented genera accounted for the remaining 2%.

### Bacterial genera identified in the GM of patients with severe UC in active state

The percentage of individual genera in the GM of patients with severe UC in active state (samples 3, 4) was also considerably different. In the uncultivated sample from patient 3 (sample 3A), we observed a higher diversity of genera compared to the cultivated counterpart (**Figure 1**). The dominant microbial genera were *Enterococcus* (21%), *Acinetobacter* (18%), *Methanothermobacter* (15%) and *Allorhizobium-Neorhizobium*-*Pararhizobium-Rhizobium* (11%). Other detected microorganisms included *Methanobacterium* spp., *Acidithiobacillus* spp., *Methanospirillum* spp., *Cloacibacterium* spp., *Corynebacterium* spp., *Lentimicrobium* spp., *Bifidobacterium* spp. and *Proteus* spp. Contrastingly, the sample cultured in SRB medium (sample 3B) showed a completely different microbial pattern with *Staphylococcus* spp. (63%), *Enterococcus* spp. (32%) and *Haemophilus* spp. (5%) making up the majority of the sample. In this case, the cultivation likely resulted in the significant increase of the *Staphylococcus* spp. and a subsequent reduction in bacterial diversity.

In sample 4, more than half of the GM of the sample without cultivation (sample 4A) consisted of the genus *Methanobacterium* (55%) with *Aliihoeflea* spp. (14%), *Methanospirillum* spp. (15%), *Acinetobacter* spp. (10%) and *Corynebacterium* spp. (7%) also being documented. The cultivation of sample 4B presumably supported the thriving of *Campylobacter* spp. which accounted for up to 84% of the detected organisms. Most of the remaining sample species were made up of *Enterococcus* spp. (13%) and *Streptococcus* spp. (2%).

### Bacterial genera identified in the GM of moderate UC patients

Regarding the samples of patients with moderate UC (**Figure 1**), sample 5 without cultivation (sample 5A) showed a high abundance of *Bacteroides* spp. (93%) and lower levels of *Escherichia-Shigella* spp. (5%) and others (2%). The sample from the same patient, but after cultivation (sample 5B), showed remarkable diversity. In particular, the dominant genera were found to be *Bacteroides* (32%), *Escherichia-Shigella* (16%), *Limosilactobacillus* (12%) and *Bilophila* (9%). Other detected genera were: *Citrobacter* (8%), *Intestinibacter* (8%), *Alistipes* (6%), *Intestinimonas* (4%), *Parabacteroides* (2%) and *Lachnoclostridium* (2%).

The most abundant genera in the sample 6 without cultivation (sample 6A) were *Bifidobacterium* (23%), *Ruminococcus gnavus* (14%), *Haemophilus* (9%) and *Methanothermobacter* (8%), while other genera represented by an abundance lower than 8% were *Lactobacillus* (7%), *Streptococcus* (6%), *Bacteroides* (3%), *Peptoniphilus* (3%), *Limosilactobacillus* (3%), *Methanobacterium* (3%), *Syntrophomonas* (2%), *Morganella* (2%), *Methanospirillum* (2%), *Longilinea* (2%) and *Lentimicrobium* (2%).

*Klebsiella* spp. (45%) and *Bilophila* spp. (12%) were predominant in the same sample, but after cultivation (sample 6B). Additional genera in sample 6B were *Bacteroides* (8%), *Cryptobacterium* (6%), *Anaeroglobus* (6%), *Morganella* (5%), *Prevotella* (5%), *Alistipes* (5%), *Streptococcus* (3%), all accounting for 8% or less.

To compare the ratios of SRB genera in the feces of all studied patients, the percentage of these microbial communities was calculated. Among all SRB, the genus *Desulfovibrio* was detected directly only in the feces of sample 2A (a patient with acute UC without cultivation). A completely different SRB genus, *Bilophila,* was revealed only after the cultivation in patients' samples 6B (patient with acute UC), 5B (patient with chronic UC) and 1B (patient with acute UC); in these patients, the genus *Desulfovibrio* was not detected.

**Figure 2:**
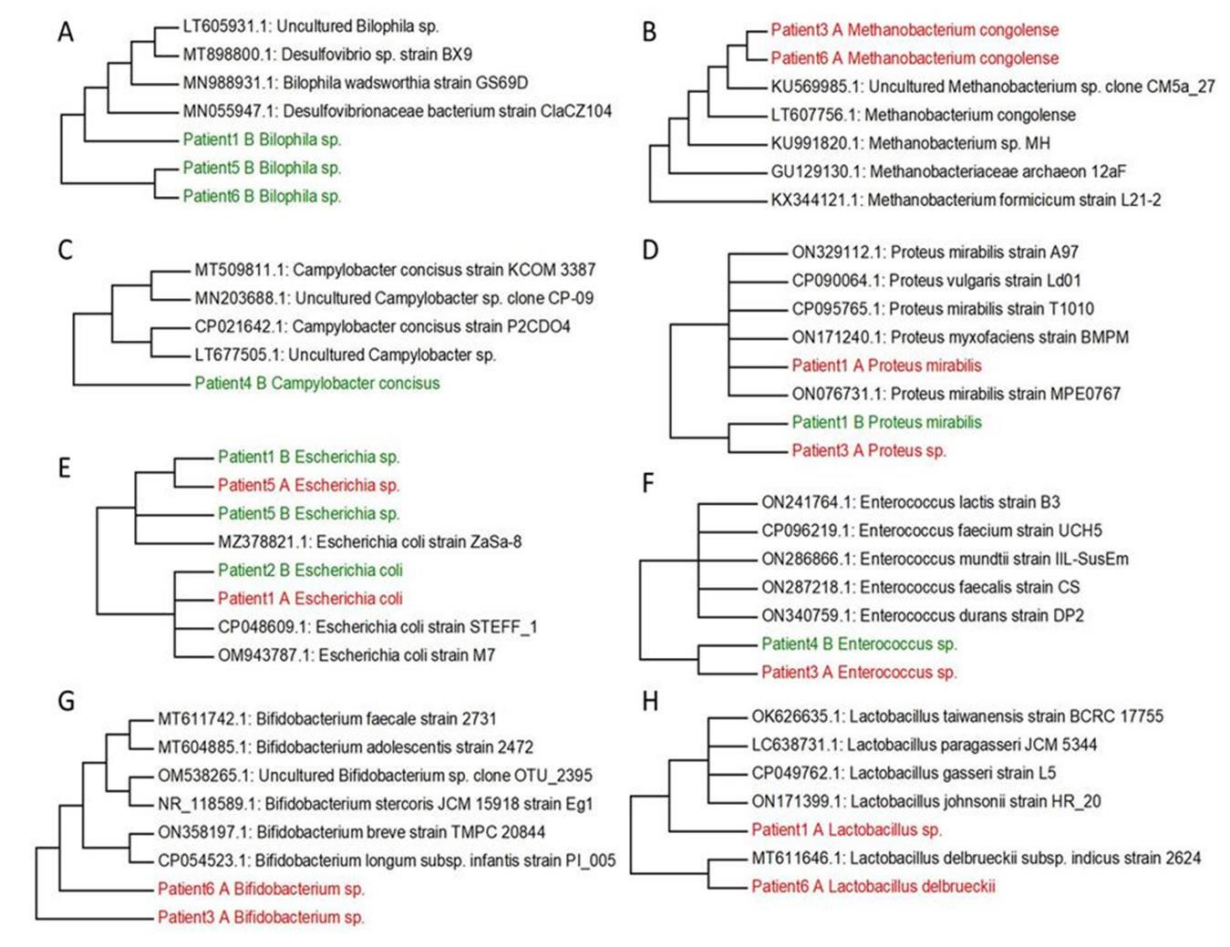
FIGURE 2: Phylogenetic trees created based on identified genera from patients with UC and the comparison of their sequences from GeneBank. *Bilophila* **(A)**, *Methanobacterium* **(B)**, *Campylobacter* **(C)**, *Proteus* **(D)**, *Escherichia* **(E)**, *Enterococcus* **(F)**, *Bifidobacterium* **(G)**, *Lactobacillus* **(H)**. Non-cultured samples are marked by red A, cultured samples are coloured by green B.

**Figure 3:**
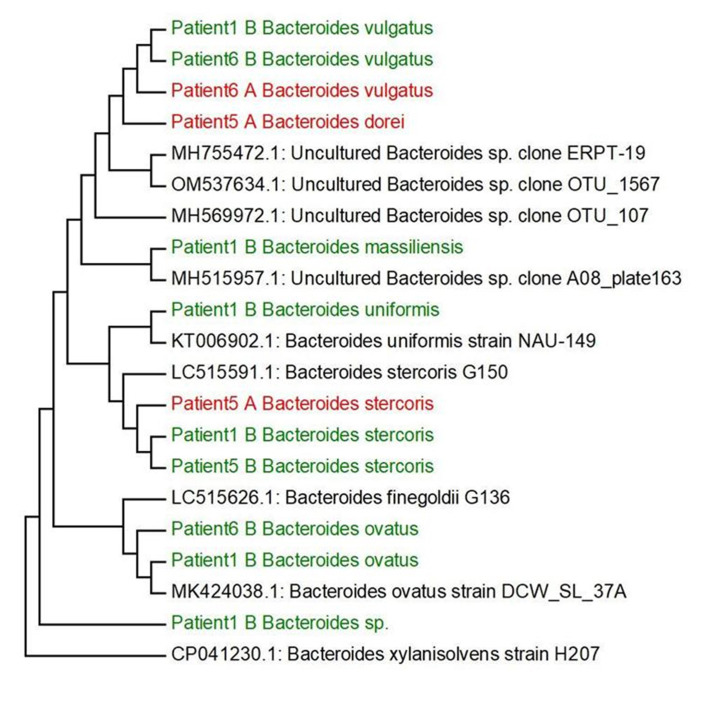
FIGURE 3: Phylogenetic trees created based on identified *Bacteroides spp.* from patients with UC and the comparison of their sequences from GeneBank. Non-cultured samples are marked by red A, cultured samples are coloured by green B.

Based on our research and research of other authors [38–44] we decided to select the main genera, that have often been associated with UC (*Bilophila, Methanobacterium, Campylobacter, Proteus, Escherichia, Enterococcus, Bifidobacterium, Lactobacterium*) and create phylogenetic trees, aiming to compare the genetic conformity of these genera in patients with severe, severe in active state or moderate UC patients (**Figure 2**). The experiment was focused on SRB which usually colonize anaerobic areas of soil, wetlands, fresh waters and marine waters, but are also known to be associated with UC development through the induction of a GM dysbiosis caused by their ability to produce high concentrations of H_2_S [5]. Recognizing the significant role of Bacteroides genus species in IBD, we opted to focus on key species within this genus, frequently linked to UC, and constructed phylogenetic trees. The goal was to compare the genetic conformity of these species among patients with severe, severe in active state, or moderate UC (**Figure 3**).

## DISCUSSION

The etiology of UC involves microbial, genetic, and environmental factors and its incidence has been increasing worldwide. Currently, the effects of UC are primarily investigated in animal models, offering various advantages. This approach allows for the exploration of the detrimental effects of this inflammatory disease and enables the assessment of the role of intestinal microorganisms in the initiation and progression of UC [45]. However, the molecular mechanisms by which gut microorganisms cause UC are yet not fully understood. Even so, a strong association between high indices of clinical activity and the presence of members of the *Enterobacterales* order (Gram-negative gut bacteria), *Clostridium perfringens* type E, *Desulfovibrio* spp. and *Enterococcus faecalis* have been found. In contrast, an association among low indices of clinical activity and *Clostridium butyricum* and *Ruminococcus albus* presence has been observed. These findings suggest that the GM composition is related to the disease severity and, furthermore, that microbial metabolites could influence UC progression. For example, SRB play a crucial role in IBD development, especially in UC, because of their ability to produce toxic compounds such as H_2_S and acetate [5, 23, 24, 34].

In general, a strong association between UC and *Clostridium difficile*, *Listeria monocytogenes*, *Escherichia coli* and *Phocaeicola vulgatus* infections have been documented [20]. Moreover, UC patients also reported a reduced number of bacterial species belonging to the phylum *Bacillota* (previously *Firmicutes*) and a considerable increase of *Actinomycetota* (previously *Actinobacteria*) and *Pseudomonatoda*, a phylum including SRB [19].

Following the cultivation of fecal samples from specific patients (1, 5, and 6) in SRB medium, only one SRB genus, namely *Bilophila* spp., was identified. The sequences of this bacterial genera formed one cluster (patient 5 and patient 6), connected with patient 1, that was genetically similar to other SRB genera, including mucin-producing *Bilophila* spp. and *Desulfovibrionaceae* members. In particular, hydrogenotrophic and mucolytic genera, such as *Desulfovibrio, Desulfobacter, Desulfobulbus* and *Bilophila* have all been associated with UC. Additionally, their secreted metabolites' mucolytic activity can be utilized by other intestinal bacteria with the consequent production of H_2_S. This in turn can directly increase gut inflammation as well as inhibit butyrate metabolism [38]. Butyric acid is a crucial SCFA with well-known and potent anti-inflammatory properties, that plays a crucial role as a histone deacetylase (HDAC) inhibitor, an energy metabolite to produce ATP and a G protein-coupled receptor (GPCR) activator. In relation to gut dysbiosis, certain studies have documented variations in the composition of the GM and the associated microbial metabolism, including differences in SCFA levels, depending on CD activity. [46, 47].

Within the *Methanobacterium* genus, *Methanobacterium congolense* formed one cluster with uncultured *Methanobacterium* sp. clone CM5a_27 and other methanogenic archaea (methanogens) (access numbers: KU569985.1, LT607756.1, KU991820.1, GU129130.1, KX344121.1). The only samples where *Methanobacterium congolense* was not detected were from samples three and six after cultivation in SRB medium. Methanogens were initially isolated in the human intestinal tract by Miller *et al.*, and they typically characterize a healthy and mature anaerobic GM [39]. However, the role and relevance of methanogens (and other archaea) in the human intestinal microbiome are still not well investigated and not yet fully understood [48]. A recent report has hypothesized that bacterial dysbiosis in the intestine of patients with IBD may contribute to an elevated abundance of methanogen species. Methanogens might potentially enhance the production of tumor necrosis factor (TNF) and activate dendritic cells, thus significantly contributing to the inflammatory state of the mucosa [49].

In general, the unresolved question still remains as to whether chronic and recurrent inflammation arises from persistent infection with a specific pathogen, overexposure to normal luminal bacterial products due to increased intestinal permeability, or an abnormally aggressive immune response to luminal components [47].

Another genus which can be isolated from the stool of patients with UC is *Campylobacter*. *Campylobacter* spp. are commensal bacteria found in cattle, sheep, pigs and birds while in humans (especially *C. jejuni* and *C. coli*) they are associated with several gastrointestinal conditions and non-intestinal manifestations including bacteremia, brain abscesses, meningitis, and reactive arthritis. Moreover, *Campylobacter* spp. and *Salmonella gastroenteritis* are associated with IBD development [40]. Only *C. concisus* was detected after the cultivation and this species has been formed with two clusters including cultured and uncultured species (access numbers: MT509811.1. MN203688.1. CP021642.1. LT677505.1)

UC patients reported the presence of *Proteus* spp*.,* a genus comprising microorganisms that has been associated with a sharp decline in absorption processes on the small intestinal mucosa. This genus may be isolated from the oral cavity, stomach, small intestinal mucosa and, most commonly, from the stool. In recent years, a primary focus has been the interrelationships between microbial quantitative and qualitative composition. This includes opportunistic pathogenic bacteria from the genus *Proteus,* which are seen to increase in patients with UC [41]. In this study, *P. mirabilis* was identified after cultivation only in patient 1, while other species of this genus have been detected in fecal samples of patient 3, but before cultivation in SRB medium. Both sequences related to a cluster, including *P. vulgaris, P. myxofaciens* and other strains of *P. mirabilis* from GenBank.

The association of *E. coli* with UC etiology has also been investigated and it has been reported that these bacteria could be found in only a small number of patients. The mucosal adhesion of these isolates was much higher compared to physiological samples or samples from patients with infectious diarrhea. The most virulent *E. coli* strains have adhesive properties, suggesting that these strains could play an impacting role in UC pathogenesis. In conclusion, there is no complete information on the role of intestinal *E. coli*, and its association with the pathogenesis of UC is controversial.

In the study conducted by Seishima *et al.* [43], metagenomic analysis of the fecal microbiota of IBD patients and fecal transplantation from responding patients to genetically susceptible animals was performed. The authors confirmed a causal relationship between inflammatory strains of *E. faecium* and UC. By sequencing multiple strains isolated from UC patients, the genotype of *E. faecium,* presumably responsible for inflammation, was identified. Thus, a causal relationship between bacterial strains and inflammation of the colon has been clearly shown [43].

As previously mentioned, patients suffering from UC have a different proportion of symbiotic or potentially pathogenic microorganisms in comparison to healthy. This difference is characterized by the expansion of members of the *Enterobacterales* order at the expense of beneficial genera, such as *Fusobacterium*, *Clostridium/Clostridioides*, *Ruminococcus*, *Lactobacillus* and *Bifidobacterium*. *Lactobacillus* spp. and *Bifidobacterium* spp. are prominent bacteria found in commercially available probiotic preparations commonly employed in the treatment of IBD. *Lactobacillus* spp. and *Bifidobacterium* spp. work to counteract the virulence mechanisms of harmful bacteria and the ensuing inflammatory response, which is closely linked to the pathogenesis of IBD. We selected these probiotic bacteria because they are an important part of the normal human intestinal microbiota; they are very well biologically characterized and are widely used in the treatment of dysbiosis [44].

A causative role for *Bacteroides* species in experimental UC was suggested in a study by Campieri and Gionchetti, (2001). In this study, the role of bacteria in UC pathogenesis was shown in animal models [50]. In a carrageenan guinea pig model of experimental colitis, germ-free animals did not develop colitis until after monoassociation with *P. vulgatus* [51]. Subsequently, it was suggested that different strains of *P. vulgatus* led to considerable differences in the inflammatory response without a correlation between the sources of strains and the severity of carrageenan-induced lesions. In this model, pretreatment with metronidazole prevented colitis, while administration of Gram-positive bacteria or coliforms was not effective. These data suggest the need for interaction between bacteria sensitive to metronidazole and dietary sulfate. More recently, the degree of cecal inflammation in HLA-B27 transgenic rats was shown to be correlated with levels of isolates on *Bacteroides* selective medium and increased anaerobic/aerobic and *Bacteroides*/aerobic ratios [52]. Indirect evidence for the interaction between the luminal microbiota and the immune system exists from studies using animal models with disruptions in immunoregulatory molecules. It was reported that spontaneous colitis, which consistently develops in knockout and transgenic murine models, does not occur when these mice are maintained in germ-free conditions [53–56]. Furthermore, it should be noted that only two genera of the *Desulfovibrionaceae* family (*Desulfovibrio* and a taurine-respiring sulfite-reducing *Bilophila*) were detected in the feces of the various patients before and after cultivation in the SRB medium. However, it should be noted that the members of the genera *Bilophila* are unable to use sulfate as an electron acceptor [57]. The species of *Bilophila* (in particular, the human stool-derived *B. wadsworthia*) caused systemic inflammation in specific-pathogen-free mice [58]. *Bilophila* members are classified as a taurine-respiring sulfite-reducing bacteria (sulfite is an intermediate reduced by dissimilatory sulfite reductase) and an H_2_S-producing bacterium [59, 60].

*B. wadsworthia*, a strictly anaerobic, sulfite-reducing bacteria and a common member of the human GM, has been associated with appendicitis and colitis [61]. H_2_S production (mostly from *B. wadsworthia*) pathways were expressed abundantly across various health states, demonstrating that these microbial functions are core attributes of the human gut [62]. Therefore, the findings documented in this study – obtained through the characterization of the GM composition of UC patients using the latest molecular methods and comparing the results with data available in publicly available databases - aimed to provide a better understanding of the course and development of this disease, paving the way for new personalized treatments of this condition in the future.

In conclusion, although UC is a chronic inflammatory condition of the colon, affecting a growing number of patients worldwide and substantially decreases their quality of life, insights can be gained from the GM of moderate and severe cases. Despite certain limitations in our study, such as the limited number and demographic heterogeneity of participants, the absence of healthy controls, the cross-sectional design, and the lower taxonomic resolution of 16S rRNA sequencing, our findings provide valuable insights into the compositional changes of the GM in patients with UC. The study presented here uses current sequencing technologies and complementary culture methods (to allow for the more effective detection of pro-inflammatory SRB) were used to identify the relevant bacterial genera in the GM of patients with UC. The results obtained do not establish a causal relationship between the presence of SRB and the onset or severity of UC. Instead, the findings highlight, among other observations, significant variations in the gut microbial composition among patients with varying disease severity and activity. Different bacterial genera were found to be dominant in each of these cases. In addition, with the use of an SRB-specific medium before sequencing, considerable changes were noted in the microbial composition of the samples (either by decreasing or increasing bacterial diversity). In particular, this increased detection of characteristic *Desulfovibrionaceae* members [68], such as *Desulfovibrio* spp. and *Bilophila* spp.

These insights contribute to a better understanding of the disease's pathogenesis and may inform the development of novel therapeutic strategies, including probiotic preparations containing beneficial bacterial communities, to counteract the virulence mechanisms of harmful bacteria and mitigate the ensuing inflammatory response.

## MATERIAL AND METHODS

### Collection of stool samples of UC patients

Patients affected by UC were enrolled at the Careggi University Hospital (Florence, Italy), after obtaining written informed consent. The description of the patients is given in **Table 1**. This study included adult (>18 years) patients affected by active UC, requiring hospitalization, while subjects were excluded if they were taking antibiotics or probiotics and similar products (prebiotics or symbiotics) four weeks before sample collection, were pregnant or lactating, were affected by other known organic gastrointestinal disease (such as, but not limited to, malignancy, chronic diarrhea; celiac disease and/or important food intolerance (e.g., lactose) and travelled to exotic areas in the last six months.

The severity of the disease has been assessed through the Baron score, the Mayo score and the Simple Clinical Colitis Activity Index (SCCAI) score.

**Figure 4:**
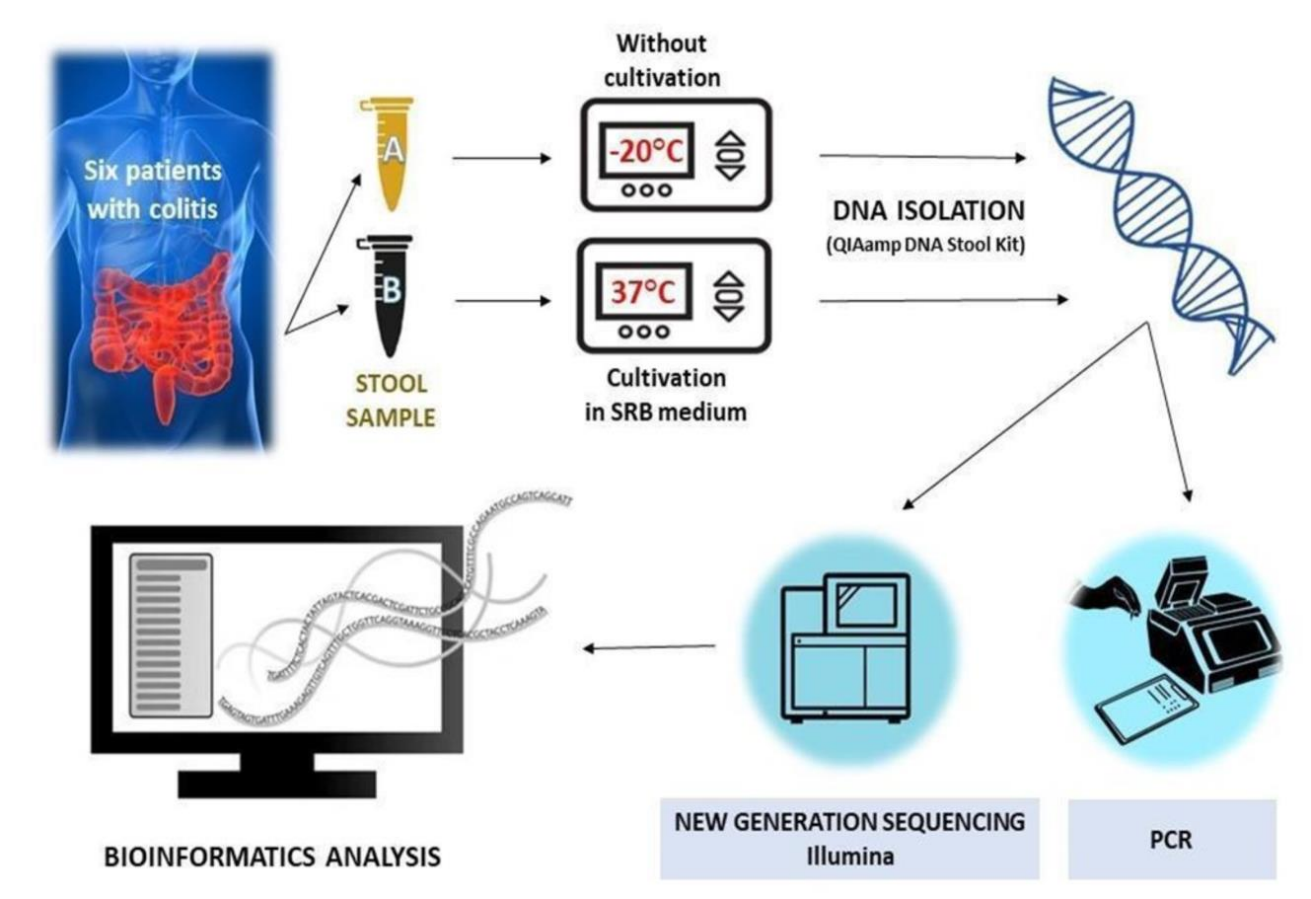
FIGURE 4: Experimental workflow without (Eppendorf tube A) or with cultivation (Eppendorf tube B) in SRB medium.

At the moment of the hospitalization, two fecal samples for each patient were collected and immediately frozen at -20°C; one was not cultivated in SRB medium, while the other was incubated in the modified SRB medium (**Figure 4**). Notably, samples 1 and 2 were taken from patients affected by severe UC, while samples 3 and 4 were taken from patients with severe UC in active state and samples 5 and 6 belonged to men with a moderate UC (**Table 1**).

All methods have been performed in accordance with the relevant guidelines and regulations included in the statement approved by the local Ethics Committee of the Tuscany Region (study 118 n°2016.0842), Careggi University Hospital and followed the principles of the Declaration of Helsinki.

### Medium for intestinal SRB cultivation

All samples for the cultivation were collected in sterile Eppendorf tubes full of the liquid medium that was bubbled with molecular nitrogen gas to attain anaerobic conditions. The composition of modified medium for intestinal SRB cultivation was as follows (grams per liter): Na_2_SO_4_ (3), KH_2_PO_4_ (0.3), K_2_HPO_4_ (0.5), NH_4_Cl (1), CaCl_2_ × 6H_2_O (0.06), yeast extract (1), sodium citrate (0.3), sodium lactate (6), MgSO_4_ × 7H_2_O (0.1), ascorbic acid (0.1) and (NH_4_)_2_SO_4_ (0.2). Potential of hydrogen (pH) range in the large intestine of humans and animals is limited (5.5–8) and it depends on many factors, including the composition and enzymatic activity of intestinal microorganisms, substrates they are able to use, the process of digestion and the quality of consumed food. As people's constant body temperature is around 37°C, cultivation was performed in a thermostat set at the same temperature, during 5 days. By respecting these conditions and creating the optimal pH and redox potential, the gut environment was simulated. This medium incorporates various organic components, thereby facilitating the growth of other anaerobic bacteria present in the human gut as well [63].

### Isolation of DNA

Total DNA was extracted using the QIAamp DNA Stool Kit (Qiagen, Hilden, Germany) from frozen (-20°C) stool samples according to the manufacturer's instructions, with minor modifications, as described below.

Firstly, 180 mg of each stool sample was mixed with 1.4 mL Buffer ASL and homogenized. The suspension was incubated at 95°C for 5 min and then centrifuged at 10.000 rpm. An InhibitEX tablet was added to the supernatant to remove impurities and PCR inhibitors. After the next centrifugation step, 200 μL of the supernatant was added to 15 μL of proteinase K solution and 200 μL of buffer AL. The mixture was incubated at 70°C for 10 min, cooled and added with 200 μL of ethanol 96%. Next, the supernatant was centrifuged through the QIAamp kit column and then washed with 500 µL of AW1 and AW2 buffers. Finally, DNA was eluted with 200 μL of AE elution buffer and stored at -20°C.

### Amplification and sequencing

Universal primers were used for the amplification of the V4 variable regions of the 16S rRNA gene [64, 65]. The primers were marked by molecular barcodes for sample identification and adapter sequences for flow cell hybridisation. Platinum™ II Taq Hot-Start DNA Polymerase (Thermo Fisher Scientific, Waltham, USA) at 0.8× was used for the PCR reaction. Cycling conditions were the following: 94°C for 3 min, followed by 35 cycles of incubation at 94°C for 45 s, 52°C for 1 min (50% thermal ramp) and 72°C for 90 s, and a final extension step at 72°C for 10 min. PCR products were purified using Agencourt AMPure XP beads (Beckman Coulter, Brea, USA), quantified and normalized using dsDNA HS assay with a Qubit 4 fluorometer (Thermo Fisher Scientific, Waltham, USA), and their quality was checked using DNF-474 HS NGS kit with Fragment Analyzer (Agilent, Santa Clara, USA).

Purified amplicons were paired-end sequenced using a Mid Output Kit (2×150 bp) with the MiniSeq platform (Illumina, San Diego, USA). Raw FASTQ reads were processed using the DADA2 package (version 1.16.0) [66], in R (version 4.0.0). Then, reads were filtered, trimmed, de-replicated and de-noised according to the standard operating procedure [67]. Afterwards, forward and reverse reads were merged, chimeras were removed, and the taxonomy was assigned by the RDP naive Bayesian classification [68] against the Silva database v138 [69].

The relative abundance of the taxonomic groups was calculated for the microorganisms detected in this study. Sequences were compared using the BLAST feature (https://www.ncbi.nlm.nih.gov/BLAST/about/) of the National Center for Biotechnology Information (NCBI) [70]. The sequences were uploaded to the Mega7 software [71] for comparative phylogenetic analyses and clustering was performed by the neighbor-joining method [72, 73].

### Data availability

The datasets used and/or analyzed during the current study are available from the corresponding author on reasonable request.

### Ethics approval and consent to participate

The patients affected by UC were enrolled at Careggi University Hospital (Florence, Italy) after obtaining informed consent and approval of the local Ethics Committee (study 118 n°2016.0842). This research was also approved by the Bioethics Committee at the Faculty of Science at Masaryk University (EKV-2021-060).

## AUTHOR CONTRIBUTION

All authors of this paper contributed, K.M., L.M. and I.K. analyzed and interpreted data from Illumina; I.K., F.G., S.B., A.A. conceptualization, methodology, and investigation of this study; I.K., D.N., S.K-M.R.R., and M.V. data curation and investigation, K.M., L.M., I.K., M.G., S.B., A.A. and M.V. writing original draft preparation, writing manuscript and editing. All authors read and approved the final manuscript.

## Abbreviations:

CD: – Crohn's disease,
IBD: – inflammatory bowel disease,
GM: – gut microbiota,
SCFA: – short-chain fatty acid,
SRB: – sulfate-reducing bacteria,
UC: – ulcerative colitis.
